# Allelic Variants of *P66* Gene in *Borrelia bavariensis* Isolates from Patients with Ixodid Tick-Borne Borreliosis

**DOI:** 10.3390/microorganisms10122509

**Published:** 2022-12-19

**Authors:** Kristina Golidonova, Eduard Korenberg, Ekaterina Krupinskaya, Vera Matrosova, Alexander Gintsburg

**Affiliations:** 1 N. F. Gamaleya National Research Centre for Epidemiology and Microbiology, 123098 Moscow, Russia; 2 Engelhardt Institute of Molecular Biology, Russian Academy of Sciences, 119991 Moscow, Russia

**Keywords:** ixodid tick-borne borreliosis, *Borrelia bavariensis*, *P66* gene, allelic variants

## Abstract

Protein P66 is one of the crucial virulence factors of *Borrelia*, inducing the production of specific antibodies in patients with ixodid tick-borne borreliosis (ITBB). Various species of *Borrelia* are characterized by genetic variability of the surface-exposed loop of P66. However, little is known about this variability in *Borrelia bavariensis.* Here we describe the variability of the nucleotide sequences of *P66* gene locus in isolates of *B. bavariensis.* Analysis of nucleotide sequences of P66 in 27 isolates of *B. bavariensis* from ITBB patients revealed three allelic variants of this gene. The alignment score of amino acid sequences in the isolates showed amino acid replacements in various positions confirming the presence of three allelic variants. Two of them are characteristic only for some isolates of *B. bavariensis* of the Eurasian gene pool from various parts of the geographic ranges of *B. bavariensis* from various samples. At least three allelic variants of *P66 B. bavariensis* have been identified, which have different amino acid expression, occur with different frequency in ITBB patients and, presumably, can have different effects on the course of the infection.

## 1. Introduction

Ixodid tick-borne borrelioses (ITBB) are chronic or recurrent spirochetal zoonotic vector-borne infections of the group Lyme borreliosis affecting various organ systems [1,2,3]. ITBB is induced by spirochetes of the *Borrelia* genus, transmitted by several species of the *Ixodes ricinus/persulcatus* complex that is common in the Northern Hemisphere [2,4,5,6,7,8]. Borrelia pathogenic for humans (*B. burgdorferi* sensu stricto, *B. garinii, B. bavariensis*, *B. afzelii, B. spielmanii*, and *B. mayonii*) [9,10,11] belong to the *Borrelia burgdorferi* sensu lato complex consisting of more than 20 species [5]. *B. bavariensis* has been described as an independent species of the genus *Borrelia* by multilocus typing (MLST) of the *Borrelia* housekeeping gene [12,13]. This species is differentiated from its closest relative *B. garinii* [13,14,15,16]. Scientific publications describe *B. bavariensis* as endemic to Asia, recently spread to Europe [17] because the European population has shown very little genetic variability [18], unlike Asian isolates [14]. In Europe, *B. bavariensis* has often been associated with neuroborreliosis [19,20,21], and in Japan it is associated with erythema migrans [15]. Besides *B. garinii* and *B. afzelii* [2,22,23] *B. bavariensis* was recently shown to be of specific epidemiological importance in the Russian Federation, often inducing an erythemic form of ITBB [9,16].

Surface membrane proteins are the crucial virulence factors of *Borrelia* [24]. These proteins are coded by genes controlling three main functions determining the survival of these microorganisms: (1) invade the vertebrate hosts (disseminate), (2) survival and development in a host, (3) and development of infectious process [25]. The chromosomal *P66* gene determines the first of these functions. It was first described by J. Bunikis [26], who suggested a topological model of the protein consisting of two intra-membrane proteins connected with a loop located at the surface of a bacterial cell. Computer models show that P66 forms a β-stem with 20–24 transmembrane and 11–12 surface domains [27,28,29]. This protein often forms oligomers [30] and belongs to the porin family with one-channel non-selective (towards anions or cations) conductance [31]. It contributes to the adhesion of the microorganism to mammalian endothelial and other cells and binds to β3 and β1-chains of integrins [32,33]. The ability of P66 to bind to integrins promotes borrelia dissemination in vertebrate hosts [34,35,36]. Enhanced expression of P66 is observed in full fed, but not unfed ticks as well as in infected experimental mammals [37,38], therefore demonstrating that the protein is probably produced when the bacteria are in a mammalian host.

P66 is used as an antigen for serological analysis of ITBB and the specific IgM and/or IgG are observed in most patients [10,39,40,41]. Immune response to P66 of B31T *B. burgdorferi* sensu stricto is directed by the surface domain of the loop, containing the epitope [42] which binds to monoclonal antibody H1337 [26,39,43]. This structure is of specific importance for the immune response [28,43,44,45,46].

Lately, the variability of the nucleotide sequences of *p66* was described in *B. burgdorferi*, *B. garinii*, *B. afzelii*, and *B. miyamotoi* [42,44,47,48,49,50,51]. However, little is known about the genetic variability in *B. bavariensis.* Here we describe the variability of the nucleotide sequences of *P66* gene locus in isolates of *B. bavariensis*.

## 2. Materials and Methods

### 2.1. Study Area

The study was performed on 27 primary isolates of *Borrelia* from patients of the Perm Regional Clinical Hospital of Infectious Diseases located in the east of Eastern Europe (58°00′50′′ N; 56°15′56′′ E). The main part of the Perm region is in the low-hill terrain of the middle Urals covered by Southern taiga. *Ixodes persulcatus* is the only vector of the causative agent of borrelioses for humans in this region. Natural reservoir hosts of these microorganisms include several species of small mammals [52,53,54,55,56]. Natural foci of borreliosis in this region contain three species of pathogenic borrelia, *B. garinii*, *B. afzelii*, and *B. bavariensis* [2,10,16,22,23]. The Perm region has the highest annual incidence of ITBB in the Russian Federation [53,54]. In 2021, it was 3.71 per 100,000 [56]. The regional features of clinical manifestations of ITBB were described previously [53,54].

### 2.2. Ethical Statement

*Borrelia* isolates were obtained in 1992 and 2006 from patients with a localized stage of the manifest form of ITBB accompanied by erythema migrans and including neurological manifestations, which manifested in some patients during their dispensary observation [56]. For seeding, completely impersonal residual biomaterials were used, provided by the attending physicians of the above Perm Regional Clinical Hospital of Infectious Diseases, which were taken from patients for diagnostic purposes. Information on the isolation of isolates from these biomaterials was published in 1998 and 2009 [54,57]. Therefore, this article uses materials obtained before the introduction of the requirement for the need for a “Statement of Informed Consent” from patients and cannot be presented by the authors at the present time.

### 2.3. Isolates of Borrelia

Bacteria from skin biopsies of the peripheral part of erythema migrans (17 isolates, Hs-1 to Hs-80) or blood plasma (10 isolates, Hs-102 to Hs-163) of patients were cultivated in BSK medium at 33 °C for one month [54,57]. Aliquots of the primary isolates stored in glycerol at −70 °C at the Borrelia Museum in the Vectors of Infections Laboratory of the N. F. Gamaleya National Research Center for Epidemiology and Microbiology of the Russian Federation Ministry of Health were used for the study [58]. The museum number of each isolate has an abbreviation “Hs” (*Homo sapiens*) indicating its source. Detailed information on the isolates was published previously [16].

DNA isolation was conducted using a commercial “PROBA-NK” kit (DNK-tekhnologia, Moscow, Russia). Previously, applying the multilocus sequence typing method using six recommended housekeeping genes and the intergenic spacer rrfA-rrlB [59], we confirmed that the studied isolates belong to *B. bavariensis* [16].

### 2.4. PCR Amplification

Polymerase chain reaction (PCR) was performed in the final volume of 30 µL. The mixture contained 1 U Tag polymerase, 10× buffer with MgCl_2_, 0.2 mM dNTP’s, 3 µL DNA, and mQ (with 0.3 µM of primers). Primers GAAATCTCAAGCTATGAAGAC (forward) and CTACATATGCTTCTGTTGAAATGG (reverse) flanking 280 nucleotide pairs of *p66* gene locus were used [45]. The forward primer was modified (C replaced by T in the position 6) using the Primer-BLAST software for better amplification of the isolates.

The PCR was done using the Eppendorf amplifier (Eppendorf, Hamburg, Germany). The reaction mixture was heated to 94 °C for 1 min, then exposed to 30 cycles of denaturation (30 s, 94 °C), annealing (30 s, 53 °C), elongation (30 s, 72 °C), and final elongation (5 min, 94 °C). Purification of PCR products (280 bp) was performed by the ethanol-acetate method and commercial kits Cleanup S-Cap (Eurogen, Moscow, Russia) and GeneJET Gel Extraction Kit (Thermo Scientific, Waltham, MA, USA).

### 2.5. Sequencing

Amplicon sequencing was conducted using the BigDye™ Terminator v3.1 Cycle Sequencing Kit (Thermo Scientific, USA). The following analysis of reaction products using forward and reverse primers was conducted using the automatic sequenator 3500xL Genetic Analyzer (Applied Biosystems, Waltham, MA, USA) of the Center of Collective Use “Genome” (Engelhardt Institute of Molecular Biology, Russian Academy of Sciences) [60]. Analysis of results was made using BLAST [61], Sequence Skanner, BioEdit, SeqMan and Jalview software. Three-parameter Tamura evolutionary model was used for nucleotide sequences, and Jones-Taylor-Thornton model was used for the amino acid sequences. Dendrograms were created in MEGA11 software using maximum likelihood method and bootstrap 1000 repeats.

### 2.6. Accession Numbers

Nucleotide sequences of the four loci representing various allelic variants of *P66* were deposited in GenBank INSDC (OP561855-OP561858).

## 3. Results

### 3.1. Analaysis of Nucleotide Sequences of p66 Gene Loci from B. bavariensis Isolates

**Figure 1:**
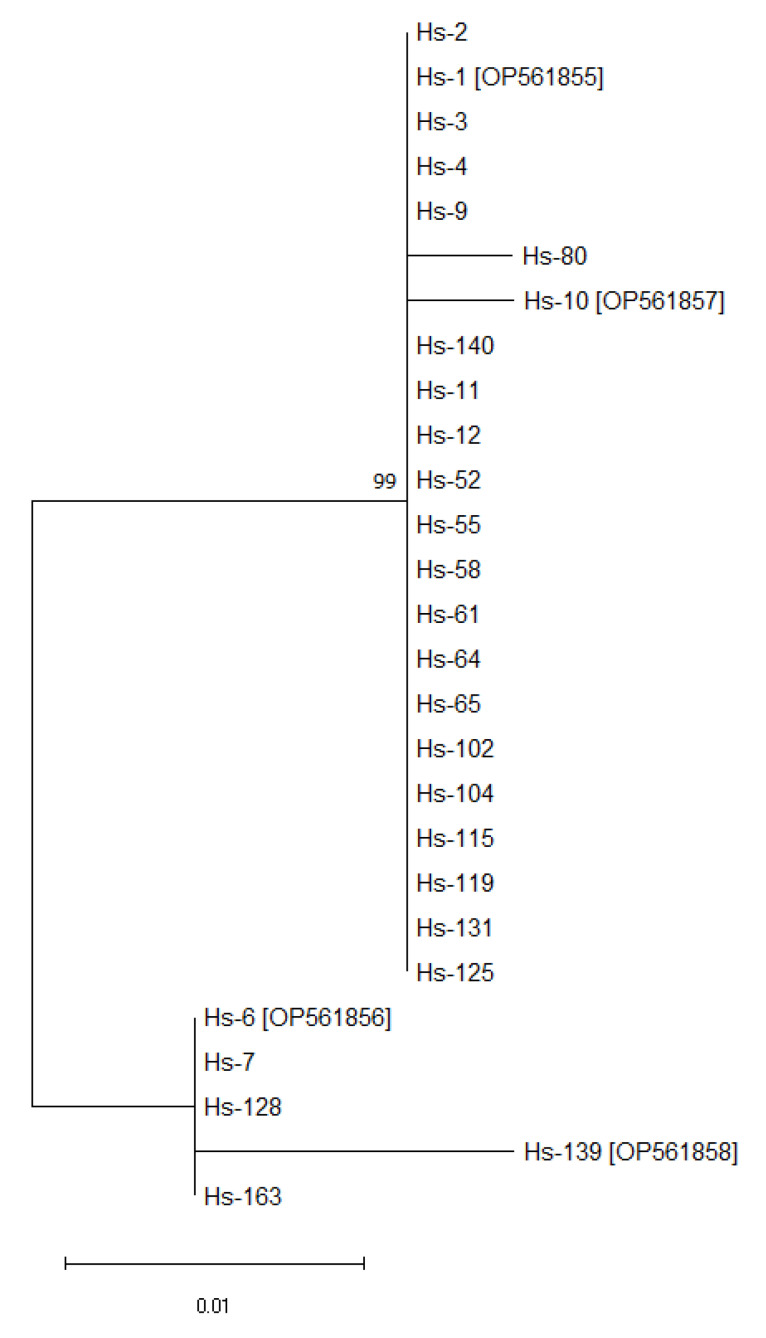
Dendrogram of nucleotide sequences of *P66* gene loci from 27 isolates of *B. bavariensis*. Created using three-parameter Tamura evolutionary model and maximum likelihood method (MEGA11 software; bootstrap 1000 repeats). GenBank numbers are between brackets.

Analysis of nucleotide sequences of *P66* gene loci of all 27 isolates from ITBB patients showed most of the sequences (20 out of 27 isolates) were identical. The remaining seven isolates differed. Hs-6, Hs-7, Hs-128, and Hs-163 were identical with each although differing from 20 identical isolates by 1.8%, and differing from isolates Hs-10 and Hs-80 by 2.1%. Isolate Hs-139 showed 96.8% minimal similarity with Hs-10 and Hs-80, and below 98.9% similarity with Hs-6, Hs-7, Hs-128, and Hs-163. The similarity between Hs-139 and the 20 identical isolates was 97.1%. Data by results indicated that various allelic variants of the nucleotide sequences of analyzed gene loci were present among the analyzed isolates. A dendrogram depicting these differences suggested that there were at least two allelic variants of *p66* (Figure 1).

**Figure 2:**
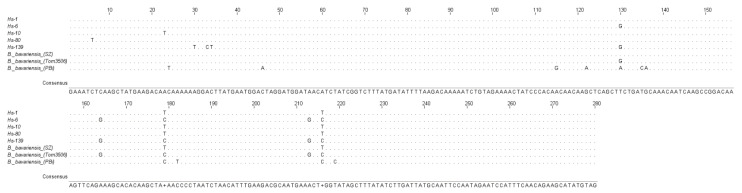
Nucleotide sequences of *P66* gene loci of *B. bavariensis* in isolates with different allelic variants. The sequences were compared with other sequences of this gene taken from the GenBank database.

One allelic variant was observed in most of the studied isolates (20 out of 27), which were isolated from samples of skin or blood plasma of patients. This group also included the sequences of the Hs-10 and Hs-80 isolates (Figure 1), which slightly differed from other isolates (one nucleotide substitution in different positions (Figure 2)).

Another allelic variant (Figure 1) was observed in four isolates (Hs-6, Hs-7, Hs-128, and Hs-163), two of which were isolated from skin samples and two from blood plasma of patients. In contrast to the first variant, the nucleotide sequences of these isolates possessed five nucleotide substitutions in similar positions (130, 164, 179, 213, 216). Moreover, we observed three additional nucleotide substitutions in positions 30, 33, and 34 in the Hs-139 isolate locus isolated from blood plasma of patient (Figure 2). These results suggested the presence of the third allelic variant of *P66* among the studied isolates.

### 3.2. Analysis of Predicted Amino Acid Sequences of P66 from B. bavariensis Isolates

**Figure 3:**
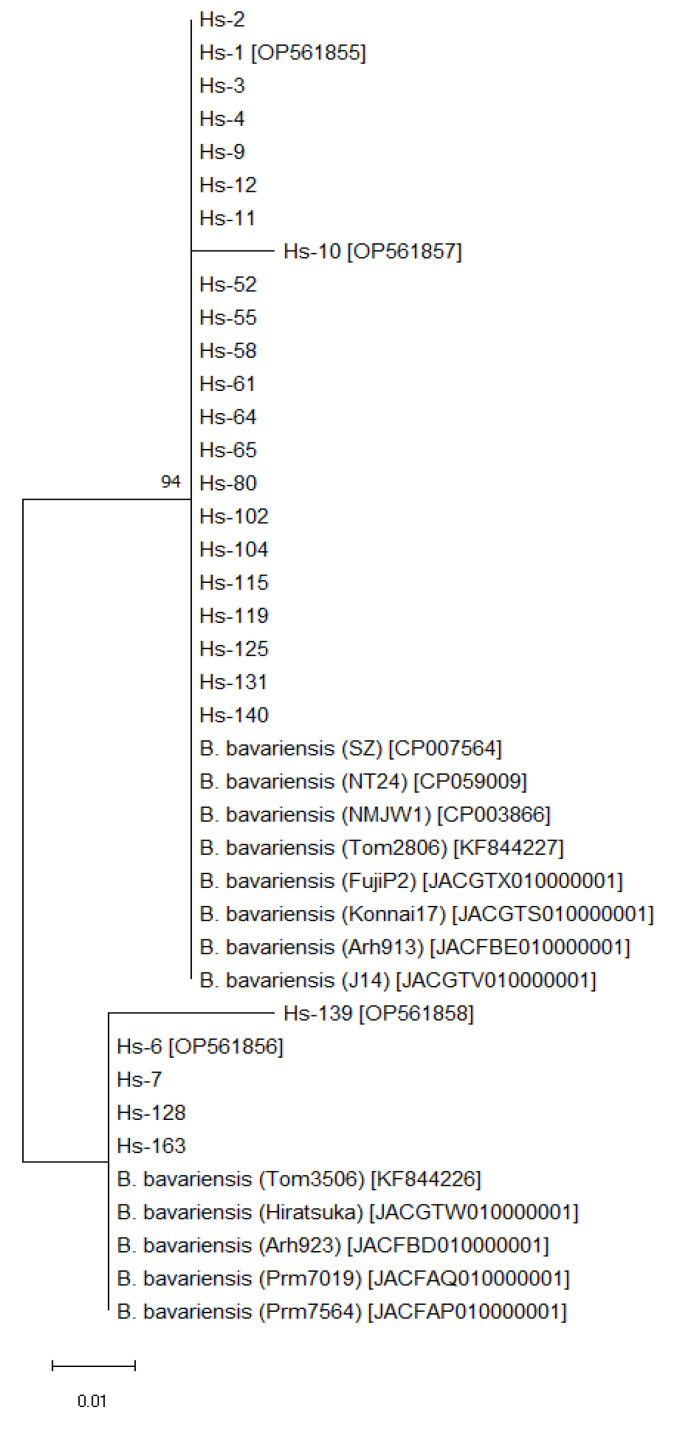
Dendrogram of amino acid sequences of P66 from *B. bavariensis* isolates. Created using Jones-Taylor-Thornton model and maximum likelihood method (MEGA11 software; bootstrap 1000 repeats). GenBank numbers are between brackets.

The nucleotide substitutions observed in *P66* gene loci could be silent or affecting the amino acid composition of the protein. This might influence the pathogenic properties of P66 in corresponding isolates. We checked the hypothesis that there are three allelic variants by creating a dendrogram of the predicted amino acid comprising the 93 residue P66 protein (Figure 3). It was very similar to the Figure 1 reflecting the presence of at least three allelic variants of *P66* gene. As shown in the Figure 1, one of them is more abundant (was observed in 22 isolates). The second variant was observed in the Hs-6, Hs-7, Hs-128, and Hs-163 isolates, and the third one only in the Hs-139 isolate. The position of the Hs-10 isolate on the dendrogram (Figure 3) allows to distinguish it even from the Hs-80 isolate characterized by maximal similarity to it (Figure 1).

**Figure 4:**
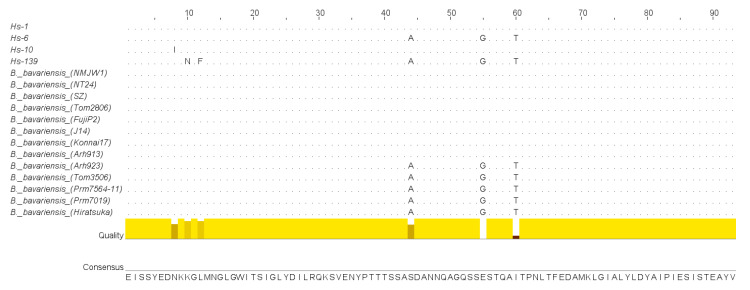
Comparison of amino acid sequences of P66 loop from various isolates of *B. bavariensis*. The quality annotation histogram reflects the likelihood of observing a mutation in any particular column of the alignment based on the BLOSUM62 matrix scores (for each column, the sum of the ratios of the two BLOSUM62 scores for a mutation pair, and each residue’s conserved BLOSUM62 score, are normalised and plotted on a scale of 0 to 1).

Presence of three allelic variants of *p66* was also supported by differences in amino acid sequences (Figure 4). The alignment score shows the amino acid sequences of P66 loop from all analyzed isolates. It also contains corresponding information on various species of *B. burgdorferi* sensu lato observed using a search with the “nucleotide collection” and “shotgun sequence” search set of databases. The alignment score variants for Hs-1, Hs-6, and Hs-139 isolates containing each of the observed allelic variants are presented in the Figure 4. Moreover, it shows the different isolate Hs-10 and similar structures of *B. bavariensis* found in the database.

Amino sequence of the Hs-6 allelic variant differs profoundly from the sequence of the major variant class (Hs-1 and others). By comparison the Hs-6 sequence has substitutions of serin to alanine (position 44), glutamic acid to glycine (position 55), and isoleucine to threonine (position 60). These substitutions might result in the changes in the configuration of the P66 loop as two polar amino acids were substituted by non-polar amino acids and vice versa.

Amino acid composition of the protein from the Hs-139 isolate additionally possessed a substitution of lysine to asparagine and leucine to phenylalanine. Thus, a polar positively charged amino acid was substituted by a polar non-charged, and a non-polar aliphatic amino acid was substituted by a non-polar aromatic amino acid (positions 10 and 12, respectively). These substitutions might significantly affect loop conformation of P66.

In the Hs-10 isolate, the amino acid sequence was very similar to it in the Hs-1 isolate. However, asparagine in the position 8 was substituted by isoleucine (polar to non-polar amino acid). These results suggest that besides the three described allelic variant of *P66 B. bavariensis*, one more variant could exist. However, this suggestion requires additional experiments.

A comparison of predicted amino acid sequences of P66 loop in *B. bavariensis* isolates we studied with those of 68 strains of this and other *B. burgdorferi* sensu lato species from the GenBank database, which had over 98% coverage, showed that they were similar only to allelic variants of *B. bavariensis* isolated in different regions from various sources, and different from sequences of other *Borrelia* species, which therefore are not shown in Figure 1, Figure 2, Figure 3 and Figure 4.

## 4. Discussion

We analyzed the genetic variability of *P66* gene locus from *Borrelia* from skin biopsies and blood plasma of the patients with acute ITBB. All studied isolates belonged to the Eurasian genetic subgroup of *B. bavariensis* [16,62]. This is, to our knowledge, the first study devoted to this species of *Borrelia*.

Gene *P66* locus consists of 280 bp and includes the sequence coding the surface domain (23–61 bp Figure 4, similar to 454–491 bp of *B. burgdorferi* sensu stricto B31T). This domain determines the immune response against P66 of *B. burgdorferi* sensu lato. Similar to *B. garinii*, the nucleotide sequence contains the AAG/AAA triplet at the position 120 (Figure 2), which is not present in *B. burgdorferi* sensu stricto B31T strain [39]. The insertion of this triplet affects the structure of the P66 loop. It determines the appearance of threonine and serine (positions 40 and 41, respectively; Figure 4) in *B. bavariensis* and *B. garinii*. Therefore, two similar species, *B. bavariensis* and *B. garinii*, are characterized by the presence of this triplet.

Three allelic variants of nucleotide sequences of *P66* and corresponding amino acid sequences were found among the studied isolates of *B. bavariensis.* These variants were observed in the isolates Hs-1, Hs-6, and Hs-139 (Figure 1 and Figure 3). The sequences corresponding to the allelic variant Hs-1 were revealed in most of the isolates and were similar to sequences from the GenBank database. These sequences are identical for *B. bavariensis* isolated from ticks *I. persulcatus* found in Russian Federation (isolate names—Arh913, and Tom2806), China (NMJW1), and Japan (NT24, FujiP2, and Konnai17), *Dermacentor* sp. from China (SZ), and a human skin biopsy from Japan (J14). The Russian Arh923, Prm7564-11, Prm7019, and Tom3506 isolated from *I. persulcatus,* as well as the Japanese Hiratsuka isolated from a human skin biopsy are similar to the Hs-6 isolate characterized by another allelic variant. However, the GenBank database does not have nucleotide and amino acid sequences, which are homologous to the Hs-139 allelic variant observed in our study. We also did not find these data for the different Hs-10 isolate, which allelic status needs to be investigated in more detail. Our results suggest that the observed allelic variants of *P66* gene locus from *B. bavariensis* isolates circulate in Eurasian natural foci of ITBB with *I. persulcatus* as the main vector. However, little is known about possible polymorphisms of this gene in borrelia in natural foci of ITBB with *I. ricinus* as the main vector.

Genetic polymorphism of *P66* gene locus was also observed in other species of *Borrelia* causing ITBB. For example, 12 allelic variants are found in *B. burgdorferi* sensu stricto [44]. *B. afzelii* is characterized by seven allelic variants, five of which were observed in the same natural foci [48,49]. Altogether, our and published data show the possibility of infection of human population with various allelic variants of vectors of ITBB.

Previous research observed five variants of P66 loop in *B. garinii* [39]. Authors of another research found only three variants of amino acid sequences in this species [51], which were analogous to ones observed in our study. Particularly, Chinese isolates IM91-13 and JC2-2 were similar to Hs-1 and Hs-6 (respectively) in amino acid positions 44, 55, and 60 (454-491 bp of *B. burgdorferi* s. s. B31T). However, as distinct from all isolates analyzed in our study, JC2-2 has a glutamic acid in the position 51 (E).

Immunology research of patients with ITBB were not the focus of our research. However, previous data showing the specific features of immune response against P66 of *B. burgdorferi* sensu lato [29,40,41,43,45,46,63] allow us to suggest that the observed variants of amino acid sequences of this protein in *B. bavariensis* might affect the immune response in patients to a certain extent. The isolates investigated in this article were obtained from humans with ITBB accompanied by erythema migrans, and like most of the isolates obtained from humans in Takano et al. [15] belong to the Eurasian subgroup of *B. bavariensis* [16], previously known as *B. garinii* NT29 [29,40,41,43,45,46,63]. Its wide distribution in natural foci of ITBB in Russia and circulation among various vectors and reservoir hosts [29,40,41,43,45,46,63,64,65] was demonstrated, and it was suggested that *B. garinii* NT29 might be the etiological agent of erythemic forms of ITBB in Russia more often than other *Borrelia* [29,40,41,43,45,46,63], whereas the European subgroup of *B. bavariensis* is often associated with neuroborreliosis [19,20,21]. It is possible that both genetic subgroups have a different course of ITBB, but this requires further research.

## 5. Conclusions

At least three allelic variants of *P66 B. bavariensis* have been identified, which have different amino acid expression, and which occur with different frequency in ITBB patients. Analysis of amino acid sequences of P66 loop of *B. bavariensis* showed the presence of hypervariable epitope, which presumably can affect the pathogenic properties of *Borrelia*, as well as have a different effect on the course of ITBB.

## Data Availability

This article does not contain any studies involving humans as primary objects of research.
